# Characterization of an L-arabinose isomerase from *Bacillus coagulans* NL01 and its application for D-tagatose production

**DOI:** 10.1186/s12896-016-0286-5

**Published:** 2016-06-30

**Authors:** Wending Mei, Lu Wang, Ying Zang, Zhaojuan Zheng, Jia Ouyang

**Affiliations:** College of Chemical Engineering, Nanjing Forestry University, Nanjing, 210037 People’s Republic of China; College of Forestry, Nanjing Forestry University, Nanjing, 210037 People’s Republic of China; Key Laboratory of Forest Genetics & Biotechnology of the Ministry of Education, Nanjing, People’s Republic of China

**Keywords:** L-arabinose isomerase, *Bacillus coagulans*, D-tagatose, Biotransformation

## Abstract

**Background:**

L-arabinose isomerase (AI) is a crucial catalyst for the biotransformation of D-galactose to D-tagatose. In previous reports, AIs from thermophilic bacterial strains had been wildly researched, but the browning reaction and by-products formed at high temperatures restricted their applications. By contrast, AIs from mesophilic *Bacillus* strains have some different features including lower optimal temperatures and lower requirements of metallic cofactors. These characters will be beneficial to the development of a more energy-efficient and safer production process. However, the relevant data about the kinetics and reaction properties of *Bacillus* AIs in D-tagatose production are still insufficient. Thus, in order to support further applications of these AIs, a comprehensive characterization of a *Bacillus* AI is needed.

**Results:**

The coding gene (1422 bp) of *Bacillus coagulans* NL01 AI (BCAI) was cloned and overexpressed in the *Escherichia coli* BL21 (DE3) strain. The enzymatic property test showed that the optimal temperature and pH of BCAI were 60 °C and 7.5 respectively. The raw purified BCAI originally showed high activity in absence of outsourcing metallic ions and its thermostability did not change in a low concentration (0.5 mM) of Mn^2+^ at temperatures from 70 °C to 90 °C. Besides these, the catalytic efficiencies (*k*_cat_/*K*_m_) for L-arabinose and D-galactose were 8.7 mM^-1^ min^-1^ and 1.0 mM^-1^ min^-1^ respectively. Under optimal conditions, the recombinant *E. coli* cell containing BCAI could convert 150 g L^-1^ and 250 g L^-1^ D-galactose to D-tagatose with attractive conversion rates of 32 % (32 h) and 27 % (48 h).

**Conclusions:**

In this study, a novel AI from *B. coagulans* NL01was cloned, purified and characterized. Compared with other reported AIs, this AI could retain high proportions of activity at a broader range of temperatures and was less dependent on metallic cofactors such as Mn^2+^. Its substrate specificity was understood deeply by carrying out molecular modelling and docking studies. When the recombinant *E. coli* expressing the AI was used as a biocatalyst, D-tagatose could be produced efficiently in a simple one-pot biotransformation system.

**Electronic supplementary material:**

The online version of this article (doi:10.1186/s12896-016-0286-5) contains supplementary material, which is available to authorized users.

## Background

D-tagatose is a natural rare ketohexose that possesses 92 % of the sweetness, but only 38 % of the calories of sucrose [1]. Since it attained GRAS (Generally Recognized As Safe) status under U.S. Food and Drug Administration (FDA) regulations, D-tagatose has become a promising functional sweetener on the food market. Until now, it has been used in the productions of confectionery, soft drinks and health foods for improving the flavors and reducing the calories. It also shows positive attributes in treatment of type II diabetes and hyperglycemia. Currently, one mature method for D-tagatose production is the direct isomerization of D-galactose into D-tagatose with metal hydroxides as the chemical catalysts under basic conditions [2]. This process was applied into commercial food grade D-tagatose production by Arla Food Company between 2002 and 2006 [3]. Nevertheless, it has been gradually dismissed because of the drastic reaction conditions and high cost of the subsequent purification steps. Another method is the enzymatic process that mainly depends on L-arabinose isomerase (AI, EC 5.3.1.4). AIs can isomerize D-galactose to D-tagatose in one step at a milder environment. The method has some significant advantages over the chemical process, such as a lower alkali dosage and less unexpected by-products [3].

**Fig. 1:**
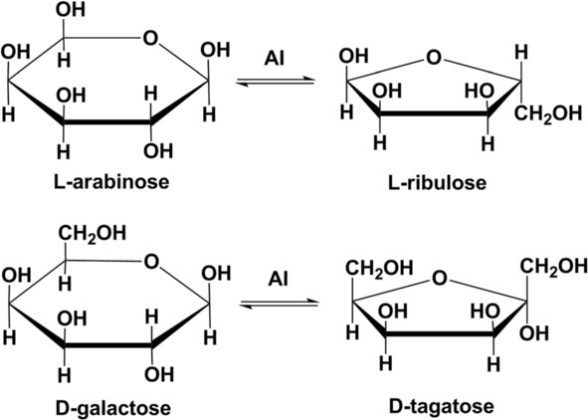
Isomerization of L-arabinose to L-ribulose and isomerization of D-galactose to D-tagatose catalyzed by AI

In microorganisms, AIs are encoded by the *araA* genes which is an important component of the L-arabinose operon and responsible for the conversion of L-arabinose to L-ribulose [4]. AIs can also recognize D-galactose and catalyze it to D-tagatose due to the similar molecular conformations of D-galactose and L-arabinose (Fig. 1). But many studies showed that the catalytic efficiency (*k*_cat_/*K*_m_) of AIs for L-arabinose was generally 10 times (or higher) than that for D-galactose and some AIs even did not show any catalytic efficiency for D-galactose [5]. Since the AI from *Lactobacillus gayonii* was presumed to be a catalyst for D-galactose isomerization in 1993 [6], about 40 types of microbial AIs have been identified and characterized so far. A large proportion of them achieved the maximal enzyme activities under moderate high temperatures (50 °C to 80 °C) and neutral-alkaline conditions (pH 7.0 to 8.0). In addition, most studies on AIs were dependent on metallic ions. Mn^2+^ and Co^2+^ always served as the activating factors of AIs and can significantly improve the enzyme activities and thermal stabilities [7]. Metallic ions in reaction mixtures are difficult to be removed in industry. Thus, their presence will reduce the purity and safety of target products.

Several reports indicated that high reaction temperature is a favorable condition for D-tagatose formation [8, 9]. Numerous AIs from thermophile strains have been reported since the year 2000 including *Thermotoga maritima*, *Thermotoga neapolitan*, *Anoxybacillus flavithermus, Bacillus stearothermophilus* US100 [8–11]. Their optimal temperatures were within 80 to 95 °C. High operation temperature causes browning reaction and formation of by-products in catalysis, which is a big obstacle for isolation of the target product [5]. Therefore, many researchers changed their interests to AIs from mesophilic *Bacillus* strains such as *Bacillus licheniformis*, *Bacillus subtilis*, *Bacillus halodurans* and *Bacillus coagulans* [12–15]. These enzymes could adapt to moderate temperatures from 50 °C to 60 °C. They possessed inherently high *k*_cat_/*K*_m_ values for the natural substrate L-arabinose. Therefore, their applications mainly focused on the synthesis of L-ribulose. Only *B. halodurans* AI was reported to show a *k*_cat_/*K*_m_ of 0.4 mM^-1^ min^-1^ toward D-galactose. Relevant data about the properties of *Bacillus* AIs in D-tagatose production are insufficient and needed to be complemented.

In this study, an *araA* gene from the *B. coagulans* NL01 was cloned and expressed in *Escherichia coli* BL21 (DE3). The biochemical properties of purified *B. coagulans* AI (BCAI) were comprehensively studied. Whole cells of *E. coli* expressing BCAI were used to produce D-tagatose under high D-galactose concentrations in order to test its actual bioconversion capacity.

## Results and discussion

### Over-expression and purification of BCAI

**Fig. 2:**
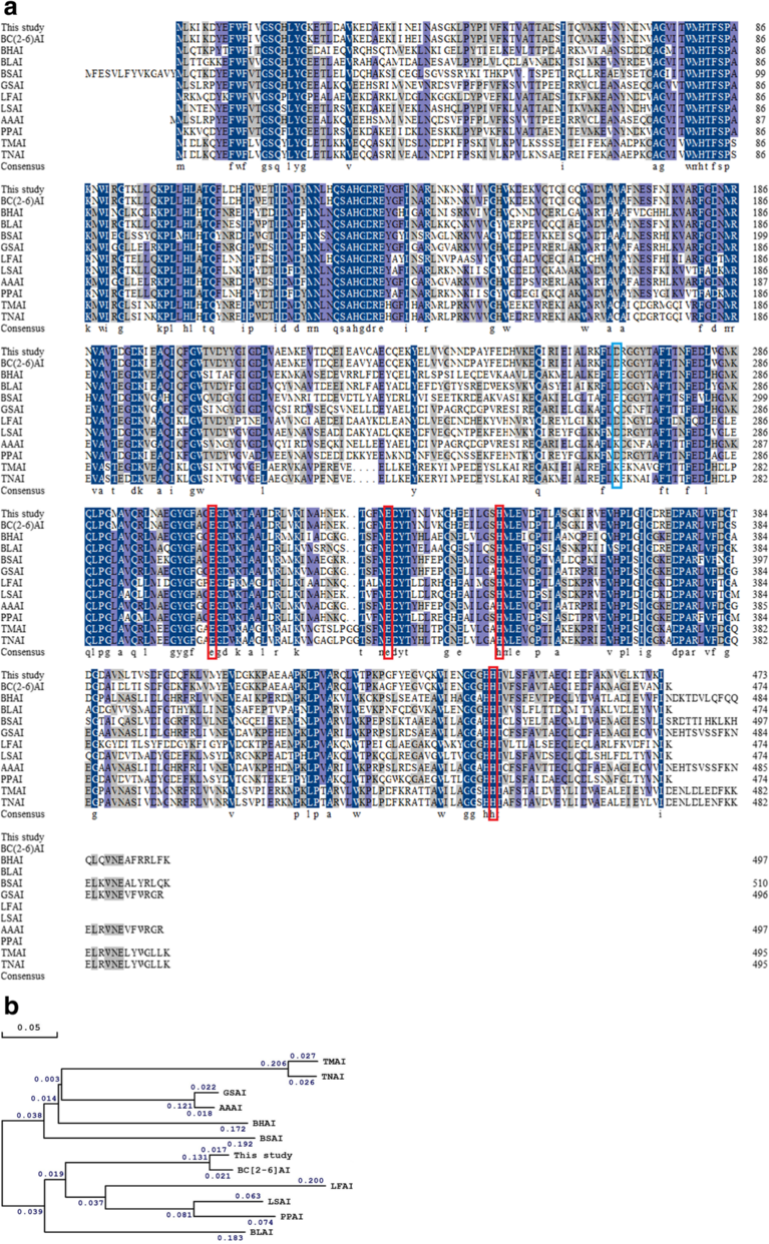
Multiple sequence alignment (**a**) and phylogenetic tree (**b**) of different bacterial AIs using DNAMAN multiple alignment tool. The amino acid sequence of BCAI (This study) was aligned with other AI sequences from different bacterial sources including *B. coagulans* 2-6 (BC [2–6]AI, AEH54185.1), *B. halodurans* (BHAI, WP_010898034.1), *B. licheniformis* (BLAI, WP_011198012.1), *B. subtilis* (BSAI, WP_003237722), *G. stearothermophilus* (GSAI, ABY84698.1), *L. fermentum* CGMCC2921 (LFAI, 4LQL_A), *L. sakei* 23 K (LSAI, YP_396468.1), *A. acidocaldarius* (AAAI, AAY68209.1), *P. pentosaceus* PC-5 (PPAI, AEM17146.1), *T. maritima* (TMAI, AKE26215.1), *T. neapolitana* (TNAI, AAK18729.1). Identical and similar amino acid residues were respectively typed *dark blue* and *purple*. The putative catalytic residues which could also bind metal ions were marked with *red boxes*. Residues that can influence the optimal pH of AIs were marked with *blue box*. The phylogenetic tree was generated using Neighbor-Joining method on the basis of the alignment above. The numbers in diagram b showed the lengths of branches that represented evolutionary relationships

The *araA* gene contained an open reading frame (ORF) of 1422 base pairs encoding a protein of 473 amino acids. Protein sequence alignments showed that, among the AIs with high activities toward D-galactose, BCAI was mostly similar to *Lactobacillus sakei* AI and *Pediococcus pentosaceus* AI with identities of 68.8 and 67.5 % respectively. By contrast, the identities to other AIs without obvious D-galactose activities from *Bacillus* strains including *B. halodurans* (57.1 %) and *B. substilis* (55.6 %) were lower (Fig. 2). Expression was induced upon addition of IPTG in Luria Bertani (LB) medium. The activity of BCAI crude extract was 2.3 U mg^-1^ for L-arabinose and 0.3 U mg^-1^ for D-galactose. Then, the crude extract was subjected to heat treatment (60 °C) and purified by HisTrap HP 5 mL column. SDS-PAGE showed distinct bands with a molecular mass around 55 kDa (expected size: 53.5 kDa, Additional file 1: Figure S1). The electrophoretically pure AI showed a specific activity of 8.0 U mg^-1^ toward L-arabinose (Table 1) and 1.1 U mg^-1^ toward D-galactose. BCAI was highly similar to the AI from *B. coagulans* 2-6 with an identity of 96.0 %, but no reports indicated that *B. coagulans* 2-6 AI possessed the same D-galactose activity [14].

**Table 1:** Purification of BCAI expressed in recombinant *E. coli*

Purification step	Protein(mg)	Total activity(U)	Specific activity(U mg^-1^)	Yield(%)	Purification fold
Crude extract	39.4	89.9	2.3	100	1
Heat treatment	15.7	78.4	5.0	87.2	2.2
HisTrap HP 5 mL column	2.3	18.3	8.0	20.3	3.5

Then enzyme activity was measured using L-arabinose as a substrate

### Effects of temperature and pH on activity of BCAI

**Fig. 3:**
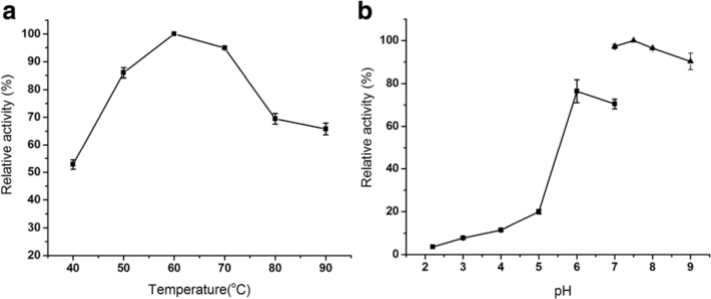
Effects of temperature (**a**) and pH (**b**) on BCAI activity. Effect of temperature was determined at pH 7.5 and effect of pH was measured at 60 °C. L-arabinose was used as the substrate. The activities at optimal temperature, pH were defined as 100 %

The effects of temperature were determined at 40 to 90 °C and pH 7.5 (Fig. 3a). BCAI displayed its maximal activity at 60 °C and retained above 85 % of the activity at 50 to 70 °C. Even at 80 to 90 °C, it still preserved above 60 % of the maximal activity. Compared with the AIs from *B. stearothermophilus* IAM11001, *Lactobacillus fermentum* CGMCC2921 and *T. mathranii*, BCAI was less sensitive to temperature change and could adapt to a broader range of temperatures [16, 17].

To investigate the effect of pH, enzyme assays were carried out at a series of pH from 2.2 to 9.0. The relative activity of BCAI reached the maximal value at pH 7.5 (Fig. 3b) and decreased by less than 10 % at pH 8.0 to 9.0. By contrast, the activity was weaker at acidic conditions. It decreased severely when the pH dropped to 5.0 as most of moderate alkaline AIs did previously, possibly because some side chain groups close to its substrate binding sites were difficult to ionize under this condition [18].

It has been experimentally proved that the optimum pH (pH_opt_) of AIs is affected by some crucial residues with polar groups, for example the E268 residue of *B. halodurans* AI (BHAI, pH_opt_ =8.0) and the equivalent D269 of *L. fermentum* AI (LFAI, pH_opt_ =6.5) [13, 17]. Modifications of the two residues to lysine (K) resulted that the pH_opt_ of BHAI and LFAI decreased to 7.0 and 5.0, respectively [19, 20]. Protein sequence alignment showed D268 in BCAI was the counterpart of E268 of BHAI and D269 of LFAI (Fig. 2a). It could be presumed that if the D268 residue was changed to lysine, the pH_opt_ of BCAI would probably decrease to a lower value.

### Effects of metallic ions on activity and thermostability of BCAI

**Fig. 4:**
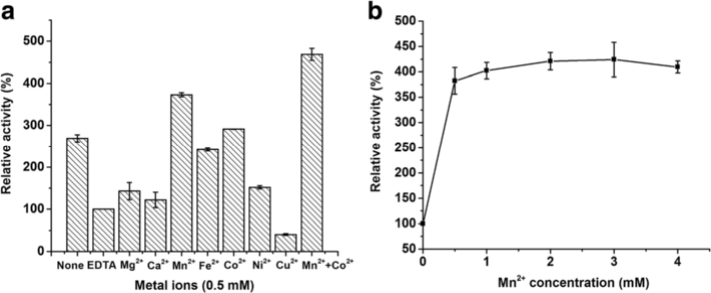
Effects of various divalent metal ions at 0.5 mM (**a**) and Mn^2+^ concentration (**b**) on BCAI activity. Enzyme assay was carried out at 60 °C and pH 7.5. None: purified BCAI without EDTA treatment; EDTA: purified BCAI treated by EDTA; The EDTA treated enzyme was used when determining of the effects of metal ions and Mn^2+^ concentration. Mn^2+^ + Co^2+^: the mixture of 0.5 mM Mn^2+^ and 0.5 mM Co^2+^

After BCAI was treated by ethylenediamine tetraacetic acid (EDTA), a dramatic loss (60 %) of activity was observed in an enzyme assay at 60 °C and pH 7.5. The EDTA-treated enzyme was then incubated in Tris-HCl (pH 7.5) solutions with different types of divalent metallic ions (Mg^2+^, Ca^2+^, Mn^2+^, Fe^2+^, Co^2+^, Ni^2+^, Cu^2+^). Enzyme assays showed that, except Cu^2+^, all other divalent ions could serve as activators (Fig. 4a). 0.5 mM Mn^2+^ and 0.5 mM Co^2+^ respectively boosted the activity by 270 and 190 % and the combination of them finally resulted in a 370 % increase. Previous researches had demonstrated that Mn^2+^ or Co^2+^ could assist AIs to transfer to correct substrate-binding conformations at elevated temperatures [21]. They significantly boosted the activities of AIs from other strains such as *T. maritima, L. fermentum* CGMCC2921 and *G. thermodenitrificans* [9, 17, 22], but some of them required higher Mn^2+^ or Co^2+^ concentrations (≥2 mM). In this study, when the Mn^2+^ concentration was increased from 0.5 mM to 4 mM, the BCAI activity changed very slightly (Fig. 4b). 0.5 mM Mn^2+^ was completely sufficient for BCAI to maintain a high activity. Further increase of Mn^2+^ by many times could not boost BCAI activity effectively but also would influence the quality of products. For purified BCAI without EDTA treatment, which will be called raw purified BCAI below, the activity was 92.6 and 72.5 %, i.e. very close to the activity of EDTA-treated enzyme measured in 0.5 mM Co^2+^ and Mn^2+^ solutions (Fig. 4a), possibly because it originally bonded metal ions or other types of ligands in the *E. coli* cells.

**Fig. 5:**
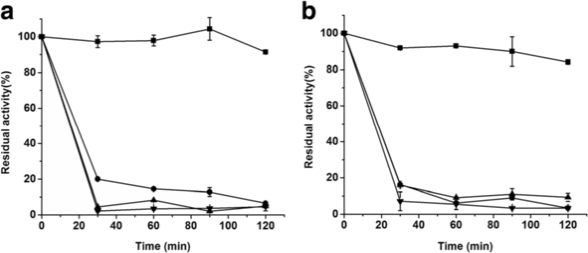
Thermal profiles of BCAI without addition of metallic ions (**a**) and in the presence of 0.5 mM Mn^2+^ (**b**). The residual activities were determined at 60 °C (■), 70 °C (●), 80 °C (▲) and 90 °C (▼). The activity of a standard reaction at 60 °C and 0 min was defined as 100 %

As EDTA treatment would not be used in practical applications of AIs, the thermal stability test was carried out using the raw purified BCAI. Figure 5a showed it was perfectly stable at 60 °C. After an incubation of 2 h at this temperature, it preserved 90 % of the initial activity. When incubated at 70 °C or higher temperatures, the enzymatic activity declined very quickly by more than 80 % during the first 30 min. Some reports showed that adding Mn^2+^ could enhance the thermostability of EDTA-treated AIs [8, 9]. But in this study, a low concentration of Mn^2+^ did not make the same enhancement effect on the raw purified BCAI. As shown in Fig. 5b, after incubation with 0.5 mM Mn^2+^ at temperatures between 60 and 90 °C for 2 h, no positive changes appeared on the residual activities of the enzyme.

From the two perspectives of enzyme activity and thermostability, it seemed that external Mn^2+^ was not essential for the raw purified BCAI. Since a low amount of metallic ions can increase the purity and safety of products, BCAI will show its unique value in food-grade D-tagatose production.

### Kinetic parameters of BCAI and molecular docking studies

Kinetic constants of BCAI were measured by following the rules of Lineweaver-Burck plots (Table 2). The catalytic efficiencies (*k*_cat_/*K*_m_) of BCAI were 8.7 mM^-1^ min^-1^ and 1.0 mM^-1^ min^-1^ for L-arabinose and D-galactose respectively. According to the ratio (8.7: 1) between the *k*_cat_/*K*m for L-arabinose and D-galactose, L-arabinose was obviously a more favorable substrate than D-galactose for BCAI. On the other hand, the *k*_cat_/*K*m for D-galactose of BCAI was noticeable because many other AIs from *Bacillus* strains had not been reported to show any catalytic efficiency for D-galactose [5, 13, 14].

**Table 2:** Kinetic parameters of AIs

Source	L-arabinose			D-galactose			
	*V*_max_ (U mg^-1^)	*K*_m_ (mM)	*k*_cat_/*K*_m_ (min^-1^ mM^-1^)	*V*_max_ (U mg^-1^)	*K*_m_ (mM)	*k*_cat_/*K*_m_ (min^-1^ mM^-1^)	
*B. coagulans* NL01	43.7	269.8	8.7	6.8	355.1	1.0	This study
*B. halodurans*	33.1	36	51	1.3	167	0.4	[13]
*B. licheniformis*	NR	369	34	NR	NR	NR	[15]
*B. substilis*	NR	120	121	ND	ND	ND	[12]
*G. thermodenitrificans*	86	142	48	NR	NR	0.5	[22]
*P. pentosaceus* PC-5	ND	ND	ND	7.8	66	2.9	[24]
*A. flavithermus*	NR	78.5	0.7	NR	25.2	5.2	[11]
*T. neapolitana*	119	116	58.1	14.3	250	3.2	[36]

Kinetic parameters of BCAI were determined by using 12.5 to 700 mM substrate (L-arabinose or D-galactose) at standard enzyme assay conditions (60 °C, pH 7.5 and 20 min)

**Fig. 6:**
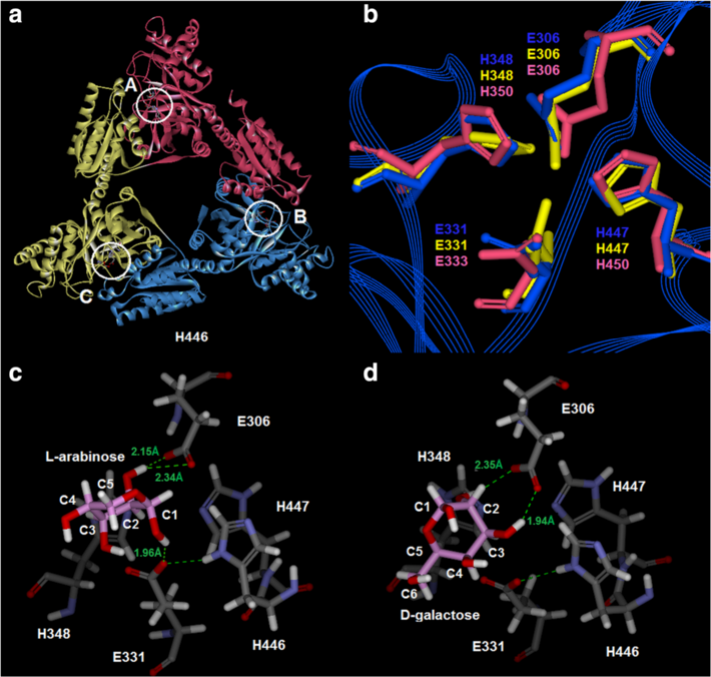
Model of *B. coagulans* NL01 AI trimer (**a**). The locations of the three substrate active sites (A,B,C) are close to the interface of two identical protein subunits. The model was constructed with the MODELLER module of Discovery Studio 4.0 package. Superimposition of the putative catalytic residues of BCAI (blue), *L. fermentum* CGMCC2921 AI (yellow, PDB ID: 4LQL) and *E. coli* AI (pink, PDB ID: 4F2D) (**b**). The complexes of BCAI active sites with L-arabinose (**c**) and D-galactose (**d**). The intermolecular H bonds are represented by green dotted lines. L-arabinose, D-galactose molecules and amino acid residues are displayed in stick form and colored according to elemental types

Molecular modelling and docking techniques were used for gaining a deeper understanding of the substrate specificity. In previous studies, the monomers of *B. licheniformis* AI (BLAI) and *P. pentosaceus* PC-5 AI (PPAI) had been comparatively modeled by using the crystal structure of *E. coli* AI as a template [23, 24]. However, the crystallographic analysis on *L. fermentum* CGMCC2921 AI (LFAI) and *E. coli* AI (ECAI) indicated that they were hexamers [25, 26]. Native-PAGE showed that the total molecular mass of BCAI was around 300 kDa, which meant that BCAI was also a hexamer (expected size: 320 kDa, Additional file 1: Figure S2). It could be assumed that substrate catalyses of these enzymes might be affected by subunit interactions. Therefore, monomer models were not accurate enough when protein subunit interactions were considered. In this study, half of the BCAI structure (a trimer, Additional file 2) was constructed on the base of LFAI (PDB ID: 4LQL, identity: 59.5 %) and ECAI (PDB ID: 4F2D, identity: 44.0 %) crystal structures. The present choice represents a compromise for achieving better conformations of the active sites and reducing computation size as well (Fig. 6a). The superimposition of the obtained BCAI structure with 4LQL and 4F2D ensured the conservation of the putative catalytic amino acids (E306, E331, H348 and H447) (Fig. 6b). The structure energy was sufficiently minimized through a 1000-step Conjugate Gradient Descent until the RMS Gradient reached 0.1.

Although molecular docking studies had been implemented on BLAI before, the result was not very representative because BLAI did not possess any D-galactose activity in experiments. Most AIs such as BCAI were able to catalyze L-arabinose and D-galactose simultaneously. As shown in Fig. 6c, two hydrogen bonds (2.15 Å, 2.34 Å) existed between the C2 hydroxyl group of L-arabinose and the oxygens of E306. One hydrogen bond (1.96 Å) was found between the C1 hydroxyl group and the oxygen of E331. According to the presumed catalytic mechanism of *E. coli* AI and *B. licheniformis* AI [26, 27], L-arabinose was firstly transformed to an enediol intermediate and then L-ribulose was formed. During the first step, protons were transferred through C1 and C2 of the substrate with the assistances of E306 and E331. The docking result here confirmed that E306 and E331 of BCAI played important roles of targeting the C2 and C1 hydroxyl groups of L-arabinose. The hydrogen bond interactions between L-arabinose and the residues were sufficiently strong. By contrast, the interactions for D-galactose were weaker. Among the two hydrogen bonds (2.35 Å, 1.94 Å) found in Fig. 6d, only the bond between the C3 hydroxyl group of D-galactose and E306 could promote the reaction according to the putative mechanism exposed above. E331 residue did not orientate the C1 hydroxyl group of D-galactose correctly, which would cause difficulties on proton transfer and slowed formation of enediol intermediate. This could be an explanation for why the formation of D-tagatose was always slower than that of L-ribulose in most AI catalyses.

Meanwhile, the C-DOCKER energies for the docking poses of L-arabinose and D-galactose were -9.39 kcal/mol and -7.07 kcal/mol respectively. A lower value indicates a more favorable binding, thus further confirming that D-galactose is poorer fit in the active site pocket of BCAI than L-arabinose.

### Conversion of D-galactose to D-tagatose by using whole cells of recombinant *E. coli*

Since BCAI could isomerize D-galactose to D-tagatose, the feasibility of D-tagatose production was further studied. It was complicated to use purified enzyme as biocatalyst in industry. Instead, whole cells of recombinant *E. coli* was constructed and selected as a suitable biocatalyst for D-tagatose production.

**Fig. 7:**
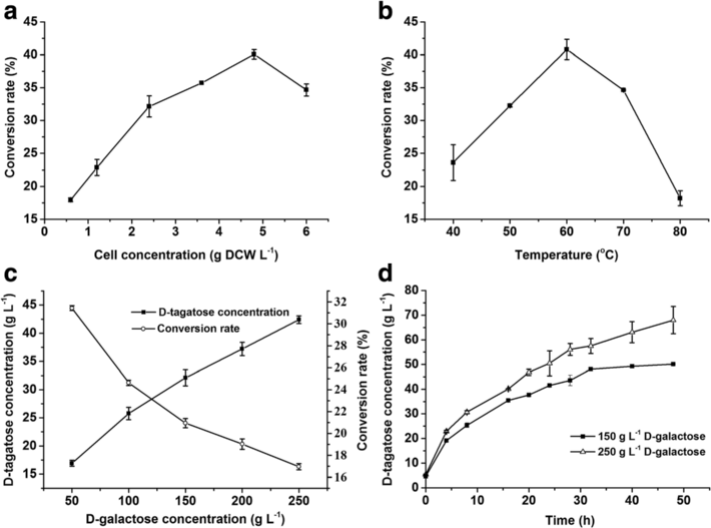
Effect of cell concentration on the conversion rate of D-tagatose (**a**). Reaction conditions: 18 g L^-1^ D-galactose, 50 mM Tris-HCl, 60 °C, 15 h. Effect of temperature on the conversion rate of D-tagatose (**b**). Reaction conditions: 18 g L^-1^ D-galactose, 50 mM Tris-HCl, 4.8 g L^-1^ *E. coli* cells, 15 h. Effect of D-galactose concentration on the conversion rate of D-tagatose (**c**). Symbols: (■) the D-tagatose concentration; (○) the conversion rate of D-tagatose. Reaction conditions: 4.8 g L^-1^ *E. coli* cells, 50 mM Tris-HCl, 60 °C, 15 h. Time course of D-tagatose conversion (**d**). Symbols: (■) 150 g L^-1^ initial D-galactose; (△) 250 g L^-1^ initial D-galactose. Reaction conditions: 150 g L^-1^ and 250 g L^-1^ D-galactose, 4.8 g L^-1^ *E. coli* cells, 50 mM Tris-HCl, 60 °C, 48 h

To increase the efficiency of D-tagatose production, the biocatalytic conditions were optimized. The effect of cell concentration on D-tagatose production was firstly investigated. As shown in Fig. 7a, the highest conversion rate was obtained at 4.8 g DCW L^-1^. Then, the effect of reaction temperature was evaluated within a temperature range from 40 °C to 80 °C. Figure 7b showed that the conversion rate reached a maximum (40.8 %) at 60 °C. It was consistent with the optimum temperature of purified BCAI. Although *E. coli* cells suffer from viability loss and cellular structure transition when the temperature is higher than 55 °C [21], these serious damages to *E. coli* cells did not suppresses the transportation of D-galactose and D-tagatose at 60 °C. The effect of substrate concentration was tested in the range from 50 g L^-1^ to 250 g L^-1^ D-galactose (4.8 g L^-1^ cells and 60 °C). With the increase of D-galactose concentration, the amount of D-tagatose kept rising without obvious substrate inhibition, whereas the conversion rate decreased from 31.4 to 17.0 % (Fig. 7c). In order to achieve a higher yield of D-tagatose, 250 g L^-1^ galactose was firstly selected for the following experiment. Meanwhile, 150 g L^-1^ D-galactose was contained, because a relatively higher conversion rate was also expected.

Based on the above experiments, the time course of tagatose production at 150 g L^-1^ and 250 g L^-1^ galactose were performed under the optimal conditions. After 32 h biotransformation, the concentrations of D-tagatose were 48.1 g L^-1^ and 55.5 g L^-1^ respectively (Fig. 7d). The conversion rates were 32.1 and 22.2 % respectively. During the next 16 h, the conversion rate for 250 g L^-1^ D-galactose rose up a little to 27.2 %. The achieved conversion rates were attractive for industrial D-tagatose production. Although immobilized AIs from *G. stearothermophilus*, *T. mathranii* and *T. neapolitana* [28–30] had been used to produce D-tagatose before, the process in this study was easier to operate and due to the enzyme purification and immobilization steps were eliminated. It was a one-pot bioconversion process and introductions of high cell density cultivation and continuous reactors could hopefully improve its feasibility in the future.

## Conclusions

In this study, an AI from *B. coagulans* NL01 was comprehensively studied. It showed a broad adaptability to moderate high temperatures. Its original dependency on added metallic ions such as Mn^2+^ was considerably low. Besides, molecular modelling of BCAI trimer combined with docking studies was used to understand its substrate specificity more deeply. Finally, a simple bioconversion system was established using whole cells of recombinant *E. coli* harboring BCAI as the biocatalyst. Attractive D-galactose conversion rates and D-tagatose productions were obtained.

## Methods

### Strains, plasmids and reagents

*B. coagulans* NL01 was stored in our lab and preserved at -80 °C [31]. *E. coli* BL21 (DE3) was used as the expression host. Plasmid pEASY-Blunt (TransGen Biotech, China) and pETDuet-1 (Novagen) were used for gene cloning and gene expression respectively. *FastPfu* DNA polymerase was purchased from TransGen Biotech (China). HisTrap HP 5 mL column was from GE Health Life Science (USA). D-galactose, D-tagatose and L-arabinose were acquired from TCI (Japan) and L-ribulose was acquired from Carbosynth (United Kingdom).

### Construction of recombinant *E. coli*

The *B. coagulans* L01 strain was cultured in LB medium for 12 h. Its genomic DNA was extracted by using TIANamp Bacteria DNA Kit (TIANGEN, Beijing) and then was used as the template DNA of PCR amplication. The primers used for cloning the *araA* gene were 5′-CGCGGATCCGATGTTGAAAATAAAAGA-3′ (forward primer) and 5′-CCGGAATTCTGTTAAAGAAGTGCATC-3′ (reverse primer). The underlined sequences represent restriction sites *Bam*H I and *Eco*R I respectively. The PCR product was ligated with pEASY-Blunt cloning vector. The resulting recombinant plasmid was sequenced by BGI Tech. (Shanghai, China). Then, both the recombinant cloning plasmid and the expression vector pETDuet-1 were digested with *Bam*H I and *Eco*R I, and the *araA* gene was cloned into the multiple cloning sites of pETDuet-1 to generate the recombinant expression plasmid, pETDuet-*araA*. Finally, the plasmid was transformed into *E. coli* BL21 (DE3) for expression.

### Overexpression of the *araA* gene and enzyme purification

The *E. coli* BL21 (DE3) harboring pETDuet-*araA* gene was grown in LB medium with shaking at 37 °C until OD_600_ reaches 0.6-0.8. IPTG was added into the medium with a final concentration of 0.5 mM for the recombinant protein expression. After incubation at 25 °C and 200 rpm for 8 h, cells were harvested by centrifugation and resuspended in phosphate buffer solution (PBS, 50 mM, pH 7.4). Cell disruption was carried out by sonication and the obtained solution was centrifugated at 10,000 × g for 15 min at 4 °C to remove insoluble cell debris. The supernatant was used as crude cell extract. For the following purification, the crude extract was heated at 60 °C in order to remove host proteins. Then it was filtered by a 0.22-μm filtering membrane and loaded on a HisTrap HP 5 mL column and equilibrated with binding buffer (20 mM sodium phosphate, 500 mM sodium chloride, and 20 mM imidazole, pH 7.4). The target protein (BCAI) was eluted with 60 % binding buffer and 40 % elution buffer (20 mM sodium phosphate, 500 mM sodium chloride, 500 mM imidazole, pH 7.4). Purity of the protein was assessed by 11.25 % SDS-PAGE. Estimation of molecular mass of multimeric protein was carried out by using 4–16 % Native-PAGE. Gels were visualized by Coomassie Blue R250 staining. The expected band size of BCAI monomer and hexamer was predicted by Compute pI/Mw tool (http://web.expasy.org/compute_pi/). Purified BCAI was stored at 4 °C for biochemical property studies.

### Enzyme assay and protein determination

The activity of BCAI was measured by determining the amount of formed keto sugar (L-ribulose or D-tagatose). Under standard conditions, 1 mL reaction mixture contained 100 mM L-arabinose or D-galactose, 50 mM Tris-HCl buffer (pH 7.5), 1 mM MnCl_2_ and 100 μL of enzyme solution at a suitable concentration. The reaction mixture was incubated at 60 °C for 20 min. Samples were cooled on ice for stopping the reaction. The amount of L-ribulose or D-tagatose was determined by cysteine-carbazole-sulfuric-acid method and the absorbance at 560 nm [32]. One unit of AI acitivity was defined as the amount of enzyme producing 1 μmol keto sugar per min under the conditions above. The protein concentration was determined by the Bradford (Sigma) method using bovine serum albumin for calibration.

### Effects of temperature, pH and metallic ion on purified BCAI

The effect of temperature on activity of purified BCAI was determined by testing the activities at temperatures from 40 °C to 90 °C at pH 7.5. The effect of pH was determined by testing the activities at pH 2 to 9 and the optimal temperature obtained above. Two buffer systems, disodium hydrogen phosphate-citric acid (50 mM, pH 2.2 to 7.0) and Tris-HCl (50 mM, pH 7.0 to 9.0) were used to get desired pH ranges.

To investigate the effect of metal ions on BCAI activity, purified BCAI was dialyzed against 50 mM Tris-HCl buffer (pH 7.5) containing 10 mM EDTA at 25 °C for 3 h. Then, the buffer was changed to 50 mM Tris-HCl (pH 7.5) for another dialysis of 36 h. Metallic ions were added into reaction mixture containing EDTA-treated BCAI at a final concentration of 0.5 mM (or 0.5 to 4 mM when studying effect of Mn^2+^ concentration). Enzyme assay was carried out at the standard condition without adding other metallic ions.

To investigate the thermostability, the raw purified BCAI was divided into two groups. One is incubated without Mn^2+^ and the other is incubated in presence of 0.5 mM Mn^2+^. The incubations are at 60 to 90 °C and pH 7.5 for 120 min. The enzyme activity was measured under the standard condition without addition of Mn^2+^.

### Determination of kinetic parameters

Kinetic parameters were determined using a 50 mM Tris-HCl buffer (pH 7.5), and 12.5 to 700 mM substrate (L-arabinose or D-galactose) and incubation for 20 min at 60 °C. The reaction was stopped by cooling on ice and the amount of L-ribulose or D-tagatose was determined by cysteine-carbazole-sulfuric-acid method.

### Molecular modelling and docking studies

The template structures for comparative modelling were searched from RCSB PDB database (http://www.rcsb.org/). The structure of BCAI trimer was constructed with the MODELLER program and validated by the Profiles-3D tool in Discovery Studio 4.0 package (DS 4.0, BIOVIA, San Diego, CA). Then the protein structure typed with CHARMm [33] force field and the substrate structures typed with MMFF94 force field [34] were subjected to energy minimizations using Conjugate Gradient Descent algorithm. Then, the substrate molecules were docked into the binding pocket of BCAI by using CDOCKER module [35]. The docking poses with the lowest interaction energy were selected for the analysis of orientation and binding interaction.

### Optimization of D-tagatose transformation conditions using recombinant *E. coli* cells containing BCAI

10 mL reaction mixtures were prepared in a 50 mL centrifuge tube containing 50 mM Tris-HCl buffer (pH 7.5). The optimal cell concentration was determined by adding 0.6 to 6 g L^-1^ recombinant *E. coli* cells to the reaction mixtures and carrying out an incubation at 60 °C for 15 h. The optimal reaction temperature was determined by incubating the reaction mixtures at 40 °C to 80 °C with the optimal concentration of *E. coli* cells. Then, a gradient of D-galactose concentrations (50 to 250 g L^-1^) were set for investigating their effect on the conversion rate. The time courses were tested with the selected D-galactose concentrations at the optimal conditions. Samples were taken periodically and analyzed by a high-performance liquid chromatography (HPLC) system (Agilent 1200 series, USA).

### Analytical methods

The amount of D-galactose and D-tagatose was measured by a HPLC equipped with a Waters Sugar-pak1 column (6.5 × 300 mm) and a refractive index detector (SHIMADZU). Deionized water was used as mobile phase at a flow rate of 0.4 mL min^-1^ and a column temperature of 80 °C [24].

## Abbreviations

AI, L-arabinose isomerase; BCAI, *Bacillus coagulans* NL01 AI; DCW, Dry Cell Weight; EDTA, Ethylenediaminetetraacetic acid; HPLC, High-performance liquid chromatography; IPTG, Isopropyl β-D-thiogalactopyranoside; PCR, Polymerase Chain Reaction

## Additional files

Additional file 1: Figure S1. SDS-PAGE analysis of the proteins from different purification steps. **Figure S2.** Native-PAGE analysis of purified BCAI. (DOCX 646 kb) Additional file 2: Sequence of BCAI trimer model. (DOCX 19 kb)
