# Supplementary material for: Characterization of an L-arabinose isomerase from Bacillus coagulans NL01 and its application for D-tagatose production
Source: BMC Biotechnol. 2016 Jun 30;16:55. doi: 10.1186/s12896-016-0286-5 (PMC4929721; doi:10.1186/s12896-016-0286-5)
Supplement: Additional file 1: Figure S1. — SDS-PAGE analysis of the proteins from different purification steps. Figure S2. Native-PAGE analysis of purified BCAI. (DOCX 646 kb) [file 12896_2016_286_MOESM1_ESM.docx]

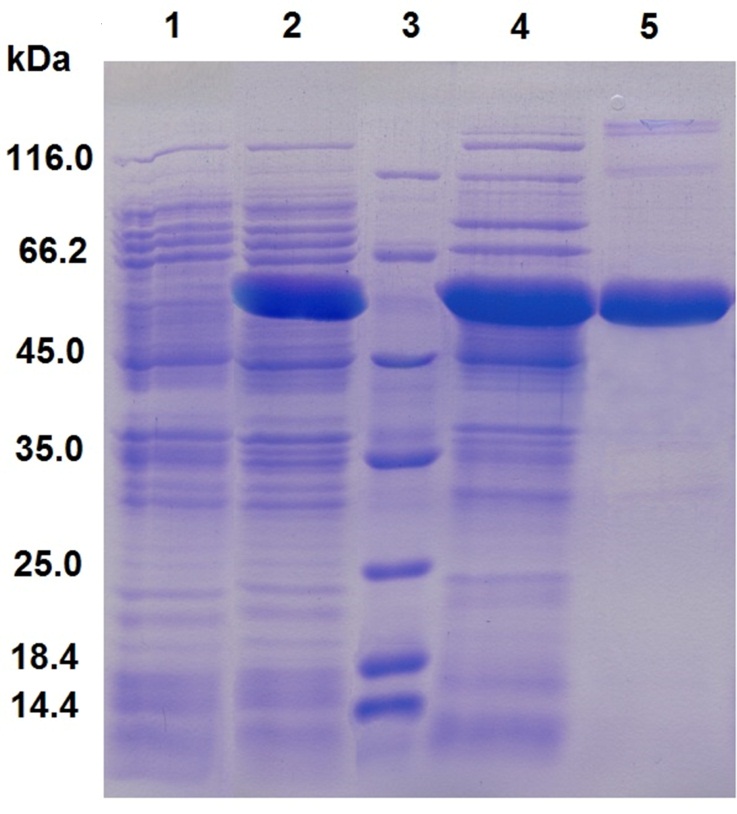


Figure S1 SDS-PAGE analysis of the proteins from different purification steps. Lane 1: negative control, crude extract of *E. coli* BL21 (DE3) cells harboring plasmid pETDuet-1; Lane2: crude extract of induced *E. coli* BL21 (DE3) containing pETDuet-*araA*; Lane 3: protein marker; Lane 4: the sample from Lane 2 after heat treatment at 60^o^C; Lane 5; the sample from Lane 4 after Ni-chelating affinity chromatography.


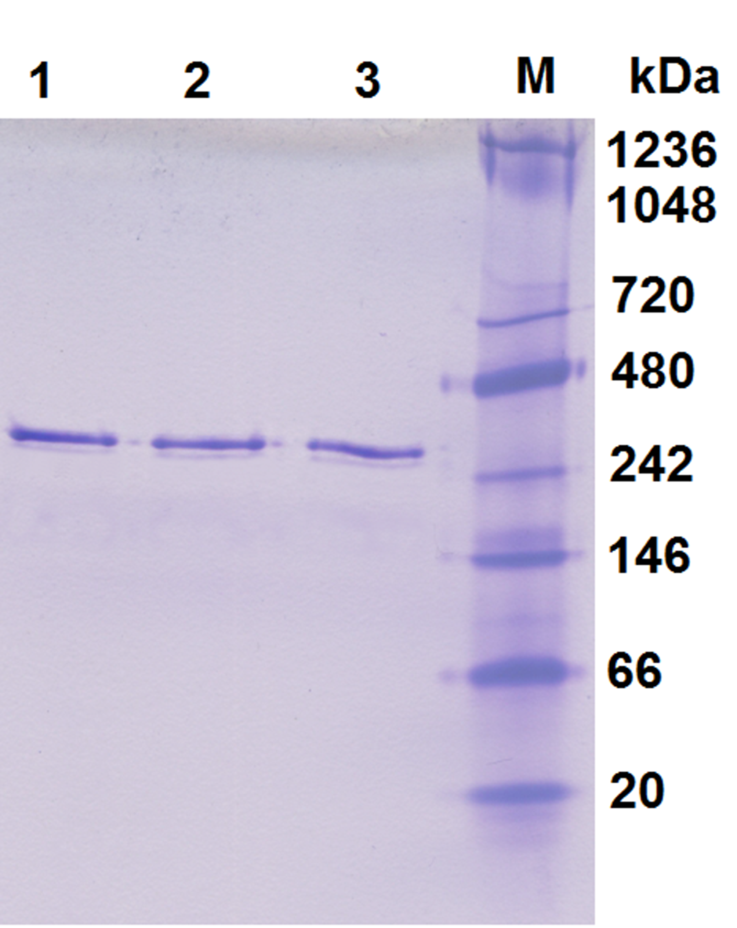


Figure S2 Native-PAGE analysis of purified BCAI. Lane 1,2,3: Non-denatured BCAI proteins; Lane M: protein standard (20 kDa-1236 kDa) purchased from ThermoFisher (USA). 4%-16% non-denaturing gel was used for the electrophoresis.

Experimental details related to Figure S2

Materials:

The seperating gel contained 12.5 M Tris-HCl (pH 6.8), 4%/0.11% - 16%/0.43% (w/v) Acrylamide/Bis-acrylamide, 0.05% (w/v) ammonium persulfate (APS) and 0.01% TEMED. The stacking gel contained 12.5 mM Tris-HCl (pH 6.8), 3.25%/0.09% (w/v) Acrylamide/Bis-acrylamide, 0.05% (w/v) ammonium persulfate (APS) and 0.01% TEMED. The (2×) sample buffer contained 8% (v/v) Glycerol, 0.125 M Tris-HCl (pH 6.8) and 0.02% (w/v) Bromophenolblue. The running buffer contained 25 mM Tris, 200 mM Glycine.

The protein standard (NativeMark™ Unstained Protein Standard) which consisted of 8 protein bands ranging from 20-1236kDa was purchased from Thermofisher (USA). Storage and use of this protein standard was according to the protocol provided by Thermofisher (https://tools.thermofisher.com/content/sfs/manuals/ NativeMarkUnstainedProteinStnd_man.pdf).

Gel running method:

1. The gradient separating gel (4% - 16% Acrylamide) was prepared in the gap between the glass plates of gel casting. The rest space was filled with water. 30-40 min was allowed for a complete gelation.
2. The water in the first step was poured out. The stacking gel was pipeted into the gap and the comb was inserted. 30-40 min was allowed for a gelation.
3. Protein sample was mixed with (2×) sample buffer (10 μl sample/ 10 μl buffer). Then, the protein standard and protein sample mixture were loaded to gel wells.
4. The running buffer was poured into the electrophoresis chamber. The electrophoresis was run at 4 ^o^C and 120 V for about 60 - 90 min.
5. The gel was immersed with staining solution containg 0.3 % Coomassie Brilliant Blue R-250 (w/v), and was shaken slowly on horizontal rotator for about 20-30min.
6. The gel was then immersed in destaining solution and put it on the same shaker for about 20-30min. The destaining solution was changed for 3-5 times until clear bands could be seen.
