# Supplementary material for: Characterization of an L-arabinose isomerase from Bacillus coagulans NL01 and its application for D-tagatose production
Source: BMC Biotechnol. 2016 Jun 30;16:55. doi: 10.1186/s12896-016-0286-5 (PMC4929721; doi:10.1186/s12896-016-0286-5)
Supplement: Additional file 2: — Sequence of BCAI trimer model. (DOCX 19 kb) [file 12896_2016_286_MOESM2_ESM.docx]

Sequence of BCAI trimer model

>BCAI trimer model

MLKIKDYEFWFIVGSQHLYGKETLDAVKEDAEKIINEINASGKLPYPIVFKTVATTADSITQVMKEVNYNDNVAGVITWMHTFSPAKNWIRGTKLLQKPLLHLATQFLDHIPWETIDMDYMNLHQSAHGDREYGFINARLNKNNKIVVGHWKDEKVQTQIGQWMDVAVAFNESFNIKVARFGDNMRNVAVTDGDKIEAQIQFGWTVDYYGIGDLVAEMKEVTDQEIEAVCAECQEKYELVVGNNDPAYFEDHVKEQIRIEIALRKFLDRGGYTAFTTNFEDLWGMKQLPGMAVQRLNAEGYGFAGEGDWKTAALDRLVKIMAHNEKTGFMEDYTYNLVKGHEEILGSHMLEVDPTLASGKIRVEVHPLGIGDREDPARLVFDGTDGDAVNLTVSDFGDQFKLVMYEVDGKKPAEAAPKLPVARQLWTPKPGFYEGVQKWIENGGGHHTVLSFAVTAEQIEDFAKMVGLKTVKI

MLKIKDYEFWFIVGSQHLYGKETLDAVKEDAEKIINEINASGKLPYPIVFKTVATTADSITQVMKEVNYNDNVAGVITWMHTFSPAKNWIRGTKLLQKPLLHLATQFLDHIPWETIDMDYMNLHQSAHGDREYGFINARLNKNNKIVVGHWKDEKVQTQIGQWMDVAVAFNESFNIKVARFGDNMRNVAVTDGDKIEAQIQFGWTVDYYGIGDLVAEMKEVTDQEIEAVCAECQEKYELVVGNNDPAYFEDHVKEQIRIEIALRKFLDRGGYTAFTTNFEDLWGMKQLPGMAVQRLNAEGYGFAGEGDWKTAALDRLVKIMAHNEKTGFMEDYTYNLVKGHEEILGSHMLEVDPTLASGKIRVEVHPLGIGDREDPARLVFDGTDGDAVNLTVSDFGDQFKLVMYEVDGKKPAEAAPKLPVARQLWTPKPGFYEGVQKWIENGGGHHTVLSFAVTAEQIEDFAKMVGLKTVKI

MLKIKDYEFWFIVGSQHLYGKETLDAVKEDAEKIINEINASGKLPYPIVFKTVATTADSITQVMKEVNYNDNVAGVITWMHTFSPAKNWIRGTKLLQKPLLHLATQFLDHIPWETIDMDYMNLHQSAHGDREYGFINARLNKNNKIVVGHWKDEKVQTQIGQWMDVAVAFNESFNIKVARFGDNMRNVAVTDGDKIEAQIQFGWTVDYYGIGDLVAEMKEVTDQEIEAVCAECQEKYELVVGNNDPAYFEDHVKEQIRIEIALRKFLDRGGYTAFTTNFEDLWGMKQLPGMAVQRLNAEGYGFAGEGDWKTAALDRLVKIMAHNEKTGFMEDYTYNLVKGHEEILGSHMLEVDPTLASGKIRVEVHPLGIGDREDPARLVFDGTDGDAVNLTVSDFGDQFKLVMYEVDGKKPAEAAPKLPVARQLWTPKPGFYEGVQKWIENGGGHHTVLSFAVTAEQIEDFAKMVGLKTVKI
